# Drivers of attractiveness and decent work in self-organizing nursing teams: a vignette study in home care

**DOI:** 10.1108/JHOM-04-2025-0178

**Published:** 2026-04-24

**Authors:** Martin Kroczek, Julia Petersen, Marcel Reiner, Ulrike Rösler, Jochen Späth

**Affiliations:** Institute for Applied Economics Research, Tübingen, Germany; Department of Nursing Science, University Hospital Würzburg, Würzburg, Germany; Federal Institute for Occupational Safety and Health, Berlin, Germany

**Keywords:** Home care, Self-organizing, Nursing autonomy, Digitalization, Job attractiveness, Working conditions, Factorial survey, Vignette study

## Abstract

**Purpose:**

Inspired by the Buurtzorg model, this study aimed to examine how the characteristics of self-organizing nursing teams and conventional job attributes affect job attractiveness and work-related strain in home care nursing.

**Design/methodology/approach:**

We conducted a standardized online survey of 98 home care nurses in Germany, including an item-based section and a factorial survey (vignette study). Hypothetical job descriptions with systematically varied job characteristics were presented, and the resulting data were analyzed using random effects models with clustered standard errors.

**Findings:**

Self-organizing team characteristics, such as nursing autonomy, flat hierarchies and participatory scheduling, did not significantly enhance job attractiveness or reduce job strain. Besides, speech-to-text documentation had a negative effect on job attractiveness and job-related strain. However, conventional job attributes, particularly workload reduction and high wages, significantly improved job attractiveness and reduced strain. Working hours had mixed effects, increasing attractiveness and physical strain. Strong heterogeneity emerged with individual factors, such as age, parental responsibilities and gender affecting job assessment.

**Originality/value:**

Our study challenges the assumption that innovative organizational models inherently improve job conditions in home care. The study results emphasize work intensity and wages as important drivers of job attractiveness and well-being, raising concerns about the premature introduction of advanced digital tools, such as speech-to-text documentation. Individualized job designs, targeted training and regulatory support are essential for fostering decent work in home care.

## 1. Introduction

Europe is aging, as demonstrated by the convergence of at least two important demographic trends for decades. First, there are significantly more old and very old people, as baby boomers are gradually getting older and Europeans are living longer, on average, than ever before (European Commission, 2020). Old age is often associated with increased multimorbidity and, consequently, increased demands for care and support (Baltes, 2003; Brijoux *et al.*, 2021; Statistisches Bundesamt, 2025; Lu *et al.*, 2025). In 2019, approximately 30.8 million people required long-term care; this number is expected to increase to 38.1 million by 2050 (European Commission, 2021). Second, declining birth rates have been observed in Europe for decades. In 2020, women had an average of 1.5 children; this is significantly below the guideline value of 2.1, which is necessary for a constant population development (European Commission, 2025). The consequence of both trends is the aging population in Europe.

Therefore, greater demands are placed on health and social care systems owing to these demographic developments (European Commission, 2024). Consequently, significant policy changes and efforts from employers are required to ensure a sustained workforce over the entire employment span.

The healthcare system as a whole, and the home care sector, in particular, are substantially affected by this challenge. The majority of older adults prefer to stay in their homes for as long as possible, and many European countries prioritize home care through policy frameworks (Genet *et al.*, 2011). In 2016, every fifth household in the EU required home-care services (European Commission, 2018). In Germany, the most populous country in the EU, more than 1 million elderly receive care at home with the support of professional healthcare workers (Statistisches Bundesamt, 2024). This makes home care the largest subsector of the country's long-term care.

In addition, working conditions in home care are demanding because of, e.g. the flexibility requirements and the necessity of working alone on private premises, which are often not structurally adapted to the needs of home care nursing (Melby *et al.*, 2018). Thus, the physical and mental strain on home care nurses is enormous, and the sickness and staff fluctuation rates have been high for years (OECD, 2020). The constantly severe shortage of home care nurses has contributed to this worsening situation.

Given these challenging conditions and their consequences, it is crucial to consider how decent work and attractive jobs in home care nursing can be promoted and ensured. One significant point of intervention lies in the optimization of working and general conditions within the framework of occupational health management. For many years, efforts have been made to improve this area at several levels. More recently, an initiative from the care sector itself has gained attention: the Buurtzorg model was developed during innovative organizational development processes in the Netherlands (Monsen and Blok, 2013). This alternative form of organizing home care is characterized by the promotion of the independence of people in need of care and the explicit involvement of extended social and local environments in the care process. According to the Buurtzorg approach, professional care teams (usually 10–12 nurses) organize themselves–from planning of working time and human resources (e.g. new hires and salary classifications) to the design and management of the care process (e.g. care planning and negotiation of activities to be performed). A small back office, advisory coaches, and training courses are available to provide administrative and professional support. Furthermore, the Buurtzorg model addresses several work-related challenges in home care. Therefore, it is a potentially promising approach to reduce work-related demands, strengthen job resources, and make home care healthier and more attractive for nurses (Laloux, 2024).

In light of the demographic shifts, the increasing demand for home care, and the persistent challenges in ensuring decent work conditions for home care nurses, there is an urgent need for evidence-based strategies to enhance the attractiveness and sustainability of employment in this sector. While innovative models such as Buurtzorg show promise, it remains unclear whether self-organizing team attributes (e.g. autonomy, flat hierarchies) are more effective than conventional job benefits in fostering health and job satisfaction in home care. Our study addresses this gap by providing empirical insights that can inform policies and practices to secure attractive and healthy work in this field.

In this study, we aimed to investigate whether the typical characteristics of self-organizing nursing teams, which constitute an important element in the Buurtzorg approach (Martela and Nandram, 2025), positively affect job attractiveness and work-related strain in home care. In addition, we sought to investigate the effect of conventional job attributes on outcomes.

## 2. Theoretical background and research model

### 2.1 Theoretical background

We conducted our analysis based on the theoretical background of the Job Demands-Resources Model (JD-R model) (Demerouti *et al.*, 2001; Bakker and Demerouti, 2007, 2018), which was established to investigate the relationships between job demands and resources and various outcomes related to employees' health and well-being. According to this model, the characteristics of any job can be divided into two spheres: demands and resources.

*Demands* are factors that require energy and effort from employees, and therefore, complicate everyday work in various ways. Demands encompass “physical, social, or organizational aspects” (Demerouti *et al.*, 2001, p. 501) and might exert negative effects on health, such as exhaustion. Furthermore, demands include the working environment and equipment, such as computers (Bakker *et al.*, 2003); for instance, computer use is a challenge for employees with fewer digital skills. Meanwhile, *resources* are factors that support task accomplishment by enabling employees to perform everyday work to reach work goals, promoting personal development, and reducing the negative effects of job demands, especially physiological and psychological costs (Bakker *et al.*, 2003). Yet, certain job characteristics may blur the boundary between demands and resources. For example, autonomy is typically regarded as a core resource that fosters motivation and engagement, and we follow this predominant view in our study. At the same time, prior research has pointed to a theoretical tension: in some contexts autonomy can also entail greater responsibility and role ambiguity, which underlines the importance of considering contextual conditions when interpreting its effects (Schaufeli and Taris, 2014).

In addition, the model primarily supports the health impairment hypothesis, which suggests that high job demands deplete individuals' resources, leading to work-related strain. By contrast, if job resources outweigh demands, it enhances employees' identification with their organization and reduces their intention to leave (motivational hypothesis).

**Figure 1:**
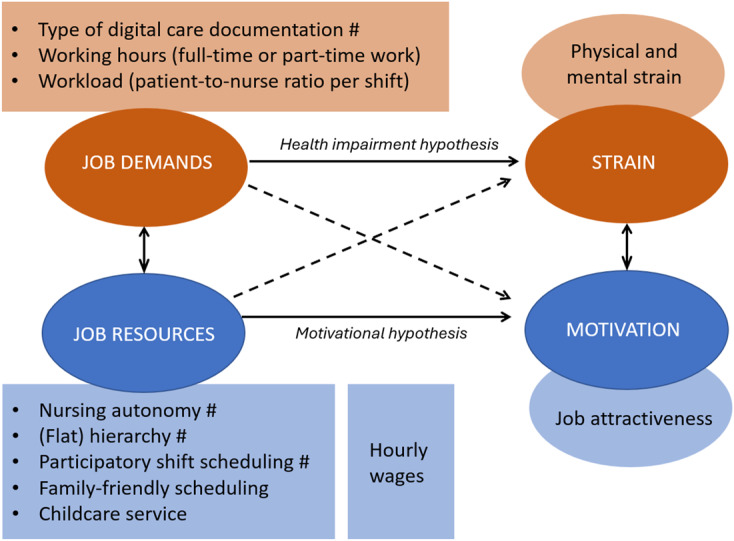
Research model based on the job demands-resources (JD-R) framework (light brown, light blue = investigated job characteristics and outcomes, ^#^ = typical job characteristics of self-organized nursing teams). Source: Authors’ own work

Our study applied the JD-R model to an empirical analysis by incorporating demands and resources as independent variables in a factorial survey of job attractiveness and work-related strain among nurses in home care (Figure 1). In addition, our analysis had another distinction. Based on the development and practical implementation of innovative organizational models in home care, we distinguished between the typical characteristics of self-organizing nursing teams and conventional job attributes.

### 2.2 Research model and research questions

A large proportion of challenges in home care services can be attributed to specific working conditions. One approach to improving work, employees' health, and the attractiveness of jobs in home care is the introduction of innovative organizational models, of which the Buurtzorg model is probably the best known. This model is characterized by a patient-oriented care system, in which the care process is based on determining individual care needs instead of a largely mechanically prescribed catalog of services to be performed (Kreitzer *et al.*, 2015; Burtke, 2018). Furthermore, in the model, there is a strong emphasis on the autonomy and personal responsibility of the nursing staff; moreover, the refinancing of the nursing staff by health insurance funds according to hours worked, instead of based on the standardized services performed, is emphasized (Kreitzer *et al.*, 2015).

The work of Buurtzorg nursing teams is characterized by a high level of digital networking, facilitating information exchange, bundling communication and bureaucracy, and a flat hierarchy (Burtke, 2018; Heer and Rosenthal, 2022). Nurses in these teams are self-organizing and make decisions regarding the care process and measures required in each case. In addition, they actively participate in organizational activities, such as scheduling and assigning clients to home care workers. Consequently, nurses have reported a high level of job satisfaction after the implementation of this concept (Burtke, 2018; Kreitzer *et al.*, 2015). In addition to the Buurtzorg model, there are other approaches to alternative organizational forms of home care services. In Germany, for example, these alternatives include “Autonome Ambulante Pflegeteams,” “Chefpfleger,” and “Pflegenachbarn” (see https://stiftung-mensch.com/neu-mook-we-gern-mit-dir/, https://chefpfleger-lenhardt.de/, and https://www.pflegenachbarn.de/).

What makes these innovative models interesting for job attractiveness and reduced work-related strain? We assumed that the Buurtzorg approach and similar approaches addressed significant demands in home care and promoted job resources, thereby strengthening the self-organization of nursing teams.

In our study, we proposed that autonomy in care delivery, flat hierarchical structures, and participatory shift scheduling represent the “typical characteristics of self-organizing nursing teams,” which are promoted by innovative care models, such as the Buurtzorg model. We also included innovative types of documentation in this category, representing innovative forms of internal communication. Furthermore, we grouped working hours, workload (patient-to-caregiver ratio per shift), family-friendly scheduling, childcare services provided by the employer, and hourly wages under “conventional job attributes.” We classified these characteristics as either demands or resources according to the JD-R model and investigated which of them better predicts job attractiveness and work-related strain. Although not captured in the JD-R model, we included pay in our study because wages are a central compensation for job strain (Rosen, 1986; Nagler *et al.*, 2025) and an important driver of job attractiveness (e.g. Doiron *et al.*, 2014; Mas and Pallais, 2017). Figure 1 summarizes our research model. It depicts how job demands (e.g. documentation, workload) are expected to increase physical and mental strain, while job resources (e.g. autonomy, participatory shift scheduling, childcare services) are hypothesized to enhance motivation and, in turn, job attractiveness. Hourly wages are included as a direct driver of attractiveness.

Several studies have investigated job attractiveness and strain in nursing, as well as the driving factors behind them (Kroczek and Späth, 2022; Hanel *et al.*, 2014; Petersen and Melzer, 2022). However, evidence on the effect of innovative work organizations in home care remains limited. To the best of our knowledge, only few studies have investigated whether the typical job characteristics of innovative organizational models in home care (i.e. self-organizing nursing teams) have beneficial effects on job attractiveness and strain or whether other factors have a decisive role. In addition, recent studies have provided empirical evidence that employee preferences for working conditions exhibit substantial heterogeneity, both generally (Maestas *et al.*, 2023; Wiswall and Zafar, 2018) and among nursing staff (Kroczek and Kugler, 2024).

Our study considers these issues and addresses the following research questions.

RQ1.
Which aspects of self-organizing nursing teams versus conventional job attributes influence job attractiveness among home care nurses?

RQ2.
Which aspects of self-organizing nursing teams versus conventional job attributes influence mental and physical strain in home care nurses?

RQ3.
Which individual characteristics moderate the relationship between working conditions and job attractiveness or job strain?

## 3. Methods and data

We conducted a standardized online survey of home care nurses in Germany. This survey consisted of two parts: an item-based section capturing individual characteristics of the respondents and a factorial survey (vignette study) to analyze the impact of working conditions on job attractiveness and strain. In factorial surveys—unlike traditional item-based surveys—several parameters are presented together within a coherent unit of meaning, rather than as separate questions. In our case, respondents evaluated an overall set of job characteristics, rather than assessing each characteristic individually. This design enables a more nuanced assessment of preferences and can reveal respondents' implicit priorities while reducing social desirability bias. For example, a person with a strong preference for high wages may rate jobs with high wages particularly highly, without having to explicitly state this (potentially socially undesirable) preference. When efficiently designed, factorial surveys also allow for causal interpretation of the effects of parameters on ratings or preference measures (Auspurg and Hinz, 2015). For these reasons, the method has been applied in various studies on job offer acceptance and attractiveness (Bähr and Abraham, 2016; Wiswall and Zafar, 2018; Kroczek and Späth, 2022).

### 3.1 Design and data analyses

In the factorial survey, we presented descriptions of hypothetical job positions in home care in text form, known as vignettes. These vignettes comprised different realizations of nine systematically varied job characteristics that are potentially relevant to job attractiveness and job-related strain (Figure 1) as dimensions. These dimensions were selected through a multistep process based on relevant literature on job attractiveness, job-related health, and self-organizing nursing teams, as well as expert discussions and pretests. By combining these dimensions into vignettes, 2^7^3^1^6^1^ = 2,304 possible vignette texts emerged. Table A1 in the Supplementary Material provides an overview of all possible vignette configurations.

To reduce the survey load, a fragmented sample of 180 vignettes was drawn from all possible combinations of vignette dimensions and distributed across different questionnaires through fragmented blocking; therefore, each respondent received 10 different vignettes. The questionnaires were randomly assigned to the respondents to ensure that the vignette variables were independent of the respondents' characteristics. The 180 vignettes were drawn from all possible combinations of vignette dimensions via D-efficient sampling, which explicitly maximizes the statistical information in the data by searching for the sample with the highest D-efficiency for a set of prespecified parameters. We applied a Resolution V design, enabling identification of all main effects of the job characteristics and all two-way interactions. Our sample achieved a D-efficiency of 97, well above the rule-of-thumb threshold of 90 for sufficient orthogonality. No pair of vignette dimensions in the final sample had a correlation coefficient exceeding 0.1.

To identify the causal effects of different vignette dimensions on job attractiveness and job-related strain, we relied on the experimental design. This allowed us to separately analyze the effect of specific dimensions. Furthermore, an introduction was added before the vignette text to prevent the influence of other unaccounted-for confounding factors on the evaluation of the vignettes. In the introduction, key confounders that may affect job attractiveness but are beyond the employer's control or not the focus of this study (e.g. the distance between home and workplace) were “controlled” by keeping them (fictionally) at a constant level across the vignettes.

After the design of the survey, it was tested using a cognitive pretest. This pretest showed that the participants had no difficulties in understanding and imagining the vignettes and dimensions in the survey. Therefore, only few linguistic adjustments were made to the final survey.

To analyze the relationship between the vignette dimensions and outcomes, we used random effects (RE) models with standard errors clustered at the individual level. As unobserved person-level heterogeneity is independent of the vignette dimensions due to the random assignment of questionnaires to the respondents, ordinary least squares (OLS) estimation would yield unbiased estimates of the causal effects of the job characteristics. However, RE estimation accounts for the two-level data structure directly and therefore produces more efficient—i.e. more precise—estimates.

### 3.2 Sampling procedure and sample characteristics

The survey addressed nurses who work or who had worked in “standard” home-care services. Former nurses could also participate if the end of their last care job was no longer than 5 years ago. Specialized care facilities, such as palliative care, 24-h care, and home ventilation, were excluded to allow the design of vignettes suitable to the–thus more homogeneous–working context. The survey was conducted in two tranches in the winter of 2023 and spring of 2024. The participants received financial incentives for completing the interview.

Nurses were recruited via advertisements and direct invitation posts on relevant Facebook groups. In addition, the invitation was sent via public mailing lists. Prior to participation, all respondents were informed about the purpose, content, and approximate duration of the survey, as well as their rights as participants. Informed consent was obtained electronically. All data were collected anonymously. Prior to the field phase, the survey was reviewed by the Ethics Committee of the Deutsche Gesellschaft für Pflegewissenschaft. Ethical approval was granted on September 5th 2023 under approval number 23–021. Participation was entirely voluntary, and individuals from vulnerable populations were not specifically targeted. All procedures complied with the ethical standards set by the reviewing institution and applicable national research ethics guidelines. By completing the survey, participants consented to the use of their responses for research purposes and for publication in anonymized, aggregated form. In total, 98 participants completed the vignettes. Not all respondents rated all 10 vignettes with regard to all outcomes, resulting in 900 rated vignettes. Table 1 summarizes the socio-demographic and organizational characteristics of our final sample. On average, participants were 45 years old, and the majority were women (78%). Around one in six had children below the age of six, and about 13% reported a foreign nationality. With respect to organizational characteristics, most participants were currently employed in home care, primarily in geriatric and healthcare services. Nearly half worked in non-profit organizations, one-third in private settings, and a smaller share in public organizations.

**Table 1:** Summary statistics of the study sample

	Mean	SD	N
*Socio-demographic variables*			
Age (years)	45.46	12.62	80
Gender (female)	0.78	0.41	79
Children below the age of six	0.16	0.37	79
Nationality			78
- German only	0.94	0.25	
- German also	0.05	0.22	
- Foreign national only	0.13	0.11	
*Organizational characteristics in present/last care job*			
Presently in home care	0.90	0.30	98
Care domain			83
- Healthcare	0.39	0.49	
- Geriatric care	0.59	0.49	
- Others	0.02	0.15	
Ownership of employer			80
- Private	0.33	0.47	
- Non-profit	0.49	0.50	
- Public	0.14	0.35	
Number of employees	244.08	1,151.95	80

**Source(s):** Survey of (former) home care staff

## 4. Results

Table 2 shows the RE estimates of the effects of the vignette dimensions on job attractiveness (column 1, research question (1) and physical and mental strain (columns 2 and 3, research question (2) expected at the described job. The dependent variables in our models were measured using an 11-point scale. Therefore, the absolute size of the coefficient of a job characteristic shows how much a job rating changes if the characteristic changes by one unit (e.g. from a less favorable to a more favorable manifestation). Wage enters the equation as a continuous variable. Figures A1 and A2 in the online supplement provide summarizing visual representations of the main effects on job attractiveness, physical strain, and mental strain and an overall overview of average and subgroup treatment effects, respectively.

**Table 2:** Main effects of the general and typical characteristics of self-organizing nursing teams on attractiveness and physical and mental strain

	(1)	(2)	(3)
	Attractiveness	Physical strain	Mental strain
Full time (40 h/week)	0.348^*^ (0.210)	0.551^**^ (0.156)	0.214 (0.178)
Wage	0.306^***^ (0.029)	−0.035 (0.022)	−0.068^***^ (0.023)
Patient-to-nurse ratio. Base: as present job	–	–	–
−2 care recipients	0.146 (0.205)	−0.546^***^ (0.158)	−0.417^***^ (0.145)
+2 care recipients	−0.989^***^ (0.206)	0.682^***^ (0.169)	0.589^***^ (0.171)
More nursing autonomy^#^	−0.084 (0.208)	0.034 (0.115)	0.099 (0.150)
Participatory shift scheduling^#^	0.086 (0.168)	−0.195^*^ (0.119)	−0.130 (0.133)
Family-friendly shift scheduling	−0.027 (0.164)	0.087 (0.121)	0.063 (0.135)
Less hierarchy^#^	0.224 (0.170)	−0.116 (0.099)	−0.295^**^ (0.123)
Alternative documentation (speech-to-text)^#^	−0.701^***^ (0.162)	0.413^***^ (0.102)	0.423^***^ (0.112)
Childcare services by employer	0.130 (0.153)	−0.206 (0.147)	−0.158 (0.131)
Constant	−1.039 (0.777)	6.204^***^ (0.588)	6.888^***^ (0.588)
Observations/vignettes	900	899	900
Individuals	98	98	98
Overall R²	0.179	0.062	0.048
Rho	0.220	0.477	0.490

**Note(s):** */**/*** indicate significance at the 10/5/1% level. The standard errors in parentheses are clustered at the individual level. Vignette dimensions and their manifestations (reference categories are underlined)

Activity; working hours: *Full time* and *50% part time*. Wage: *six wage levels*. Number of patients per nurse: *as present job*, *− 2 care recipients*, *+ 2 care recipients*. Autonomy: *fixed service catalog (lower autonomy)* and *no fixed catalog (higher autonomy)*. Participatory shift scheduling: *dictatory scheduling* and *participatory scheduling*. Family-friendly shift schedule: *No specific family-friendly schedule* and *family-friendly shift schedule*. Hierarchy: *clear hierarchy* and *less hierarchy*. Type of care documentation: Smartphone and speech-to-text. Childcare: *No childcare services provided by the employer* or *childcare services provided by the employer*. ^#^ = Typical job characteristics of self-organizing nursing teams

**Source(s):** Factorial survey of (former) home care staff, own calculations

Speech-to-text documentation is the only innovative organizational characteristic that affects *job attractiveness*, with a coefficient of −0.70 (*p* < 0.01). This effect size is substantial, corresponding to almost three quarters of a scale point–comparable to the effect of a wage increase by two euro per hour. The finding suggests that respondents exhibit marked preferences against this elaborated digital form of documentation, indicating that, in practice, introducing such tools could meaningfully reduce the perceived attractiveness of home care jobs.

Regarding conventional job attributes, we found that full-time and better-paid jobs were significantly more attractive than part-time and lower-wage jobs. Job attractiveness decreased with increasing workload, that is, when nurses needed to care for more patients in the advertised job than in their present job. Two particularities emerged regarding workload. First, the effect of workload was not monotonic. An increase in the number of patients per shift compared to their present job showed a significant effect but a decrease in the number of patients had no effect. Second, the effect of workload was considerably large. An increase of two additional patients per shift reduced job attractiveness by almost one point (β = −0.99, *p* < 0.01) on the 11-point scale–exceeding the impact of a three euro per hour wage increase and of all other job characteristics in our study. This highlights the central role of workload in shaping job preferences.

The effects on *physical and mental strain* partially matched the results for attractiveness. Speech-to-text documentation was, again, perceived negatively. The innovative form of documentation increased both physical and mental job strain, with coefficients of 0.41 (*p* < 0.01) and 0.42 (*p* < 0.01), respectively, and would nearly offset a decrease in job strain from a reduction in the care ratio by two patients per nurse. In contrast, participatory shift scheduling reduced physical strain (β = −0.20, *p* < 0.10). Finally, jobs in more hierarchical organizations were associated with higher mental strain (effect of less hierarchy: β = −0.30, *p* < 0.05). Together, these findings highlight that while innovative documentation can inadvertently heighten strain, participatory organizational practices can alleviate it.

Regarding conventional demands and resources, full-time jobs were perceived as more attractive than part-time jobs (β = 0.35, *p* < 0.10) but also as more physically demanding (β = 0.55, *p* < 0.05). Workload showed a clear dose-response pattern: each increase of two additional care recipients raised both physical (β = 0.68, *p* < 0.01) and mental strain (β = 0.59, *p* < 0.01), whereas reductions in workload lowered strain accordingly. Finally, higher wages were associated with reduced mental strain (β = −0.07, *p* < 0.01), underlining that compensation not only improves attractiveness but also might reduce perceived demands, albeit with a considerably lower effect.

To investigate *individual heterogeneities* within the relationship between working conditions and our investigated outcomes (research question 3), we analyzed the effects of the vignette dimensions and the three subgroups of respondents. Tables 3-5 present the results of this analysis. The tables are differentiated with respect to age, along the median of the age distribution, whether a respondent was living with children below the age of 6 years, and the gender of the respondent. Most effects were similar across subgroups. However, we observed differences for certain combinations of dependent variables, job characteristics, and individual characteristics.

**Table 3:** Group average treatment effects on job attractiveness, categorized by age, children below the age of 6 years in the household and gender

	Average effect	Age		Children <6 years		Woman	
		Low	High	No	Yes	No	Yes
Full time	0.348*	0.469	0.038	0.180	0.632	1.092**	0.034
Wage	0.306***	0.293**	0.37***	0.334***	0.256***	0.347***	0.316***
−2 care recipients	0.146	0.283	−0.265	−0.041	1.214***	0.015	0.072
+2 care recipients	−0.989***	−1.09**	−1.15***	−1.161***	0.01	−0.502	−1.187***
Higher autonomy	−0.084	0.276	−0.082	0.129	0.212	−0.26	0.118
Participatory roster	0.086	0.273	0.034	0.313	−0.414	0.371	0.061
Family-friendly roster	−0.027	−0.002	−0.006	−0.103	0.343	−0.431**	0.071
Less hierarchy	0.224	0.141	0.498*	0.406**	−0.267	−0.302	0.351*
Alternative documentation (speech-to-text)	−0.701***	−1.128***	−0.382*	−0.681***	−1.443**	−0.480	−0.832***
Childcare services provided	0.130	0.194	0.065	−0.072	1.04***	0.116	0.104
*N*	900	400	400	660	122	170	612

**Note(s):** */**/*** indicate significance at the 10/5/1% level. Standard errors are clustered at the individual level. Vignette dimensions and their manifestations (reference categories are underlined)

Activity; working hours: *Full time* and *50% part time*. Wage: *six wage levels*. Number of patients per nurse: *as present job*, *− 2 care recipients*, *+ 2 care recipients*. Autonomy: *fixed service catalog (lower autonomy)* and *no fixed catalog (higher autonomy)*. Participatory shift scheduling: *dictatory scheduling* and *participatory scheduling*. Family-friendly shift schedule: *No specific family-friendly schedule* and *family-friendly shift schedule*. Hierarchy: *clear hierarchy* and *less hierarchy*. Type of care documentation: Smartphone and speech-to-text. Childcare: *No childcare services provided by the employer* or *childcare services provided by the employer*

**Source(s):** Factorial survey of (former) home care staff, own calculations

**Table 4:** Group average treatment effects on physical strain, differentiated by age, children below the age of 6 in the household and gender

	Average effect	Age		Children <6 years		Women	
		Low	High	No	Yes	No	Yes
Full time	0.551**	0.369*	0.618*	0.596***	0.330	0.290	0.628***
Wage	−0.035	−0.049*	−0.059*	−0.030	−0.140***	−0.088*	−0.059**
−2 care recipients	−0.546***	−0.688***	−0.444	−0.543***	−0.988**	−0.444	−0.613***
+2 care recipients	0.682***	0.623**	0.857***	0.753***	0.043	0.786*	0.668***
Higher autonomy	0.034	−0.207	0.068	−0.036	−0.479***	0.288	−0.137
Participatory roster	−0.195*	−0.435**	−0.059	−0.288**	−0.110	−0.578**	−0.178
Family-friendly roster	0.087	−0.039	0.225*	0.204*	−0.345	0.136	−0.016
Less hierarchy	−0.116	−0.043	−0.100	−0.067	−0.046	0.332	−0.140
Alternative documentation (speech-to-text)	0.413***	0.353***	0.495***	0.342***	0.820***	0.083	0.534***
Childcare services provided	−0.206	−0.113	−0.306	−0.057	−0.796**	−0.116	−0.135
N	899	400	400	660	121	170	611

**Note(s):** */**/*** indicate significance at the 10/5/1% level. Standard errors are clustered at the individual level. Vignette dimensions and their manifestations (reference categories are underlined)

Activity; working hours: *Full time* and *50% part time*. Wage: *six wage levels*. Number of patients per nurse: *as present job*, *− 2 care recipients*, *+ 2 care recipients*. Autonomy: *fixed service catalog (lower autonomy)* and *no fixed catalog (higher autonomy)*. Participatory shift scheduling: *dictatory scheduling* and *participatory scheduling*. Family-friendly shift schedule: *No specific family-friendly schedule* and *family-friendly shift schedule*. Hierarchy: *clear hierarchy* and *less hierarchy*. Type of care documentation: Smartphone and speech-to-text. Childcare: *No childcare services provided by the employer* or *childcare services provided by the employer*

**Source(s):** Factorial survey of (former) home care staff, own calculations

**Table 5:** Group average treatment effects on psychological strain, categorized by age, children below the age of 6 in the household and gender

	Average effect	Age		Children <6		Women	
		Low	High	No	Yes	No	Yes
Full time	0.214	0.208	0.034	0.251	−0.152	−0.032	0.227
Wage	−0.068***	−0.083***	−0.086**	−0.063***	−0.123**	−0.144***	−0.075***
−2 care recipients	−0.417***	−0.670***	−0.232	−0.424***	−1.021**	−0.641***	−0.510***
+2 care recipients	0.589***	0.479*	0.930***	0.758***	−0.084	0.438	0.725***
Higher autonomy	0.099	−0.139	0.098	−0.073	−0.083	0.214	−0.038
Participatory roster	−0.130	−0.297*	0.013	−0.169	−0.192	−0.277	−0.166
Family-friendly roster	0.063	−0.075	0.208	0.117	−0.157	0.098	−0.016
Less hierarchy	−0.295**	−0.240*	−0.339*	−0.375***	0.124	0.084	−0.343**
Alternative documentation (speech-to-text)	0.423***	0.600***	0.399**	0.446***	0.690	0.210	0.608***
Childcare services provided	−0.158	−0.123	−0.213	−0.124	−0.326	−0.255	−0.092
N	900	400	400	660	122	170	612

**Note(s):** */**/*** indicate significance at the 10/5/1% level. Standard errors are clustered at the individual level. Vignette dimensions and their manifestations (reference categories are underlined)

Activity; working hours: *Full time* and *50% part time*. Wage: *six wage levels*. Number of patients per nurse: *as present job*, *− 2 care recipients*, *+ 2 care recipients*. Autonomy: *fixed service catalog (lower autonomy)* and *no fixed catalog (higher autonomy)*. Participatory shift scheduling: *dictatory scheduling* and *participatory scheduling*. Family-friendly shift schedule: *No specific family-friendly schedule* and *family-friendly shift schedule*. Hierarchy: *clear hierarchy* and *less hierarchy*. Type of care documentation: Smartphone and speech-to-text. Childcare: *No childcare services provided by the employer* or *childcare services provided by the employer*

**Source(s):** Factorial survey of (former) home care staff, own calculations

Regarding job attractiveness, speech-to-text documentation and changes in the number of care recipients had a significant effect for all groups except for men. Older nurses, women, and nurses without young children viewed a less hierarchical organization positively. Childcare provision had a significantly positive effect only for nurses with young children.

Regarding physical job strain, speech-to-text documentation was considered to lead to more physically demanding work in all subgroups, except for the relatively small subgroup of men. Participatory scheduling lowered the physical demand rating for younger nurses, nurses without children, and men only. Full-time work was not perceived as more physically by men or nurses with children below the age of 6 years. Family-friendly rosters were perceived to increase physical strain by older participants and those without young children, and jobs with employer-provided childcare were rated less demanding by nurses with young children, only.

Regarding psychological strain, speech-to-text documentation was considered to increase psychological strain in all groups, except in the group of nurses with children below the age of 6 years and men. Less hierarchical organization was not perceived to decrease mental strain by nurses with young children and men. Participatory scheduling decreased the mental demands only for younger nurses.

## 5. Discussion

We investigated how the typical demands and resources of self-organizing nursing teams, compared to conventional job attributes, affect job attractiveness and mental and physical strain among home care nurses. In addition, we evaluated the role of individual characteristics in moderating these relationships.

We found that the characteristics of self-organizing teams did not appear to be of decisive importance in promoting job attractiveness or reducing job-related strain. Enhanced nursing autonomy through service catalogs with less rigid specifications, flat hierarchies, and participatory scheduling did not show compelling effects with regard to more attractive or less demanding jobs in home care. Speech-to-text documentation had a significantly negative effect on outcomes, whereas conventional job attributes showed positive effects. This applies in particular for a reduction in workload and promotion of resources in the form of better payments. The role of working hours was ambivalent; while working hours had a positive effect on attractiveness, they had a negative effect on physical strain.

At first glance, our findings appear to contradict previous findings to some extent. Autonomy is a widely acknowledged job resource, beginning with early studies by Karasek and Theorell (1990). Accordingly, Scott *et al.* (2015) found that nurses value autonomy, and Kroczek and Späth (2022) identified autonomy as a factor that enhances job attractiveness. Estryn-Behar *et al.* (2010) reported that lack of autonomy increases nurse turnover. Fields *et al*. (2018) highlighted that nurses value a strong nursing voice in management, which may indicate their intention to participate in the decision-making process. The concept of magnet hospitals, founded in the USA in the 1980s, promotes participation-oriented organizational culture and presently leads in care quality and employee satisfaction (see Anderson *et al.*, 2018 for an integrative review). Why do the typical characteristics of self-organizing nursing teams have no effect in our data? There are two potential explanations.

First, we suspect that the advantages of self-organizing nursing teams do not come to fruition because the demands are still too high and therefore exceed resources substantially. Workload in home care has been a significant problem for decades (Cœugnet *et al.*, 2016; Guo *et al.*, 2019). The Covid-19 pandemic further exacerbated this situation, particularly because of increased work intensity (Petersen *et al.*, 2024). In this regard, our results seem to agree with previous findings that the buffer hypothesis postulated by Karasek and Theorell (1990) and Bakker *et al.* (2005) in the JD-R model cannot be confirmed universally across different contexts and populations (Fall *et al.*, 2015; Pidgeon *et al.*, 2014). This interpretation is further supported by findings from AL‐Abrrow *et al.* (2021), who showed that nurses in Iraq rated their jobs as less attractive during the COVID-19 pandemic–despite presumably unchanged core job structures. This suggests that under extreme pressure, workload can overshadow other, potentially positive, job characteristics.

Second, the respondents in our study may not have felt adequately trained to have more autonomy (and responsibility), actively participate in organizational processes, and use elaborate digital technologies in everyday nursing, as advertised in our hypothetical job descriptions. This points to a potential challenge within the JD-R framework already indicated in the theoretical section (Schaufeli and Taris, 2014): while job autonomy is typically conceptualized as a valuable resource–as we did in our study–it may paradoxically function as a stressor when employees perceive a mismatch between their skills and the demands associated with autonomous decision-making. Evidence from occupational health research supports this view, showing that job autonomy, usually regarded as a resource, can under certain conditions be associated with non-linear or even adverse effects on strain and health (Bradtke *et al.*, 2016). In such cases, autonomy might not alleviate strain but rather amplifies feelings of overload. Referring to our study results, we therefore suspect that transferability of the Buurtzorg model may be questionable because nurses in the Netherlands, in contrast to nurses in Germany, have had academic education for many years and, therefore, may be better equipped to meet the requirements of innovative organizational models. Likewise, Hegedüs *et al.* (2022) concluded in their review of the implementation of the Buurtzorg model outside the Netherlands that the transformation from a previous, highly hierarchical, division of labor-based care work to a holistic approach that emphasizes (and requires) nurse autonomy is challenging. Comparable evidence from research on Magnet hospitals and Buurtzorg-inspired nursing models supports this interpretation. Current studies show that translating Magnet principles into existing organizational cultures–supported by collaborative structures and professional development opportunities–is essential for lasting change (Nurmeksela *et al.*, 2026; Svensson *et al.*, 2024). Without such supportive conditions, hierarchical traditions and limited nursing autonomy may impede the establishment of empowerment-oriented practices. Similarly, analyses of Buurtzorg adaptations in the United Kingdom or Brazil highlight substantial implementation barriers due to hierarchical management traditions (Bernstein *et al.*, 2022; Lalani *et al.*, 2019). These findings align with our results, suggesting that the effectiveness of self-organizing models depends on contextual conditions, including professional education and socialization, organizational culture, and readiness for participatory structures. In the German context, limited exposure to autonomous team models and persistently high workloads may restrict the realization of such benefits. However, the situation in Germany may change in the coming years. Since nursing education was reformed in 2017, stronger academization has been observed. In the future, this could create more favorable conditions for implementing innovative care models like Buurtzorg or other forms of self-organizing teams, provided that these are accompanied by appropriate organizational and political framework conditions (Büscher *et al.*, 2022).

Two other findings merit discussion. Hourly wage had a strong and positive effect on job attractiveness and perceived strain, whereas speech-to-text documentation had a negative effect.

The finding that an increase in hourly wages increased job attractiveness and reduced perceived job-related strain, suggests a potentially important role of this attribute and is broadly consistent with previous research (Hanel *et al.*, 2014; Kankaanranta and Rissanen, 2008; Fields *et al.*, 2018). As stated by Herzberg *et al.*, 1959, salary is a hygiene factor, an important prerequisite for job satisfaction (Herzberg *et al.*, 1959). However, Herzberg also postulated that only motivational factors, such as recognition, responsibility, and opportunities for development foster true job satisfaction and long-term motivation. This explains research findings that nurses are willing to sacrifice part of their salaries in exchange for improved working conditions and a more positive work environment (e.g. Kroczek and Späth, 2022; Scott *et al.*, 2015; Song *et al.*, 2015). In recent years, wages in the German care sector have increased. The minimum wage for care assistants was raised to €15.50 per hour in 2024, and a further increase is planned. In addition, the median wage in the nursing profession has increased at a disproportionately high rate (Carstensen *et al.*, 2024). However, wages vary depending on the region and employer, and home care nurses continue to earn lower wages than nurses in other care settings (Carstensen *et al.*, 2024).

Speech-to-text documentation had an overarching negative effect, possibly because elaborate digital technologies in home care are not yet state of the art and may, therefore, not be essential requirements in job choices. Moreover, the introduction of highly elaborate applications, such as speech-to-text documentation, might be considered a stress factor that tends to discourage nurses, regardless of age, family situation, or gender. While our vignette-based results point to potential challenges in the acceptance of digital tools, these findings should be interpreted with caution given the hypothetical nature and limited size of our study. Rather than firm prescriptions, they provide tentative indications that supportive regulatory specifications, carefully developed digitization strategies within home care services, time for testing and on-the-job improvements, tailored training, and human-centered, ergonomic technology design may prove useful in addressing such concerns (Seibert *et al.*, 2023; Toghian Chaharsoughi *et al.*, 2022; Wolf-Ostermann and Rothgang, 2024).

In the subgroup analyses, we examined how the interdependencies between job characteristics and job attractiveness, as well as physical and mental strain, differed across relevant groups. While most effects were similar across subgroups, we also observed some indicative differences related to individual characteristics. Specifically, the drivers of job attractiveness varied by age group, gender, and childcare obligations, consistent with previous research on job preferences in nursing (Kroczek and Kugler, 2024) and, more generally, in the labor market (Mas and Pallais, 2017; Maestas *et al.*, 2023; Wiswall and Zafar, 2018). We also found differences between these groups in what they perceived as physically and mentally demanding. Given the small and partly unbalanced subgroup sizes, these findings should be interpreted as exploratory rather than conclusive. Nevertheless, they provide suggestive evidence that tailoring jobs in home care to individual characteristics and preferences could help reduce perceived job strain and increase attractiveness for targeted subgroups. Rather than a one-size-fits-all approach, differentiated or individualized job designs—in which tasks are aligned with individual skills, interests, and needs—may be more effective (Lawler and Finegold, 2000).

One of the strengths of our study is its methodological approach (factorial design), which combines the advantages of experiments and surveys by creating controlled but realistic scenarios (vignettes). This allowed the investigation of an innovative organizational model whose usage is currently still not very widespread in home care practices. Our vignettes simulated hypothetical and at the same time realistic job situations so that respondents can make authentic decisions. Furthermore, a high level of experimental control was maintained because the variables can be systematically varied. Nevertheless, the decision-making situations presented in our vignettes remain hypothetical, and the respondents' answers may not coincide with their behavior in comparable real-life situations with actual consequences, which may limit the external validity of our results. Future studies should investigate whether our findings can be confirmed in real-life settings.

Furthermore, some more specific limitations should be acknowledged. First, our sample comprises only about 100 respondents and was obtained through online recruitment. This constrains the external validity of our findings due to the small sample size, the sampling frame, and the potential for self-selection bias. Second, our operationalization of self-organization necessarily reduced the holistic Buurtzorg model to selected dimensions. This simplification may have limited observed effects, as it neglects the model's broader cultural and professional embeddedness. However, the chosen dimensions emerged from expert discussions as the most salient and practically relevant in the German home care context and enabled us to translate otherwise abstract concepts into experimentally tractable scenarios. Thus, our study does not test the Buurtzorg model in its entirety but examines proxies of self-organization, offering a first empirical step where large-scale field studies are not yet feasible due to the limited presence of Buurtzorg-type nursing teams in Germany. Third, we assessed job attractiveness and strain using single 11-point items rather than validated, multidimensional scales. While this choice is consistent with the logic of vignette experiments–where participants are asked to evaluate hypothetical scenarios rather than their own lived experiences–it restricts construct validity, which could have attenuated the observed relationships and might, in consequence, drive non-significant results. Our findings should therefore be seen as relative evaluations across conditions, and future research on self-organizing nursing teams could employ multidimensional measures to validate and extend our results. Moreover, future methodological research employing multi-item measures in separate survey designs could help assess the robustness of null findings in factorial surveys that rely on a single outcome variable more broadly. A fourth point concerns our conceptualization of workload variation via the number of patients per nurse. Nursing workload varies not only along the extensive margin but also along the intensive margin; that is, nursing complexity may differ across patients or cases. To measure nursing complexity, it is often operationalized through the number of nursing diagnoses and actions (e.g. Cesare *et al.*, 2025; Cocchieri *et al.*, 2025), which are particularly salient in hospital settings. In contrast, in home care, complexity tends to arise from the interplay of chronic conditions, psychosocial needs, and long-term coordination across multiple actors. Our vignettes were designed to abstract from variation in case-related complexity by specifying that the expected effort per patient remains constant. Although the experimental setup of our survey is robust to changes in the set of dimensions—as long as all relevant features are either included as dimensions or held constant in the descriptions—the effect of workload could differ in a design where workload varies not only with the number of patients (extensive margin) but also with the workload per patient (intensive margin). Consequently, the interpretation of effect sizes for other dimensions (e.g. characteristics of self-organizing teams) could change in relative terms due to changes in the relative effect size (i.e. when compared to work load).

## 6. Conclusion

Given the nursing shortage in Europe, improving working conditions and job attractiveness is urgent. The World Health Organization (2025) calls for person-centered care models, and innovative approaches like self-organizing teams may help meet this goal. However, our findings suggest their implementation is challenging and must be accompanied by reduced workload and strengthened resources. Future research should compare models of self-organizing nursing teams across countries to understand the preconditions for successful transfer.

While our findings provide relevant insights, they should be interpreted with caution due to certain limitations—most notably the hypothetical nature of the scenarios and the small, non-representative sample. Nevertheless, they highlight several practical and policy implications: First, although central characteristics of self-organizing teams, like autonomy and participation in management, can be important job resources, an effective strategy to improve attractiveness and reduce the mental and physical strain of home care work should foremost focus on conventional job characteristics. A manageable caseload and adequate staffing regulations can support retention, while higher wages would help attract and keep nursing staff, and even after significant increases in recent years, wages in home care are persistently relatively low, indicating room for action. Second, the adoption of innovative forms of care organization should account for, and potentially adapt to, contextual factors, especially employees' competencies and readiness for participatory structures. Self-organization may appeal less to the still overwhelmingly occupationally trained nursing workforce in Germany. However, as nursing in Germany becomes more professionalized and academic, preferences for more autonomy and greater participation in management may increase. And which is more, in line with previous research and research on other occupations, we find nurses' preferences to be heterogeneous, meaning that some nurses may particularly benefit from alternative care structures, while others do not. Overall, a differentiation of working characteristics can possibly better suit individual preferences. Last, the introduction of new, more efficient digital technologies is not necessarily viewed with optimism by potential users in outpatient care. Where new technologies are introduced, these should therefore not only be tested extensively and tailored to care workers' needs, but also, they should be accompanied by a comprehensive change management process–just like the switch to more self-organizing teams. Such processes require attention to both structural and human dimensions of change. Effective change management should not only ensure that new tools and forms of collaboration are meaningfully integrated into daily routines, but also foster understanding, trust, and ownership among staff. This includes transparent communication of objectives and benefits, active involvement of care workers, and continuous support through training, mentoring, and reflection. Despite the modest scope of the (average) effects of self-organizing team characteristics in our study, it is premature to dismiss models like Buurtzorg. Of course, change management appears to be only a necessary but not sufficient condition for their successful transfer to other countries and nurses. While our findings point to several important implications for implementing innovative care models, further research using workplace-level or participatory approaches would be needed to derive more concrete guidance for day-to-day practice.

## Supplementary Material

Data supplement 1

## Notes

The supplementary material for this article can be found online.
