# Supplementary material for: Drivers of attractiveness and decent work in self-organizing nursing teams: a vignette study in home care
Source: J Health Organ Manag. 2026 Apr 24;40(9):325–44. doi: 10.1108/JHOM-04-2025-0178 (PMC13153437; doi:10.1108/JHOM-04-2025-0178)
Supplement: Data supplement 1 [file jhom-04-2025-0178_suppl1.docx]

**Online supplement**

Table A I. Vignette universe and related outcome questions

| Dimension | | | Level | | Vignette Text | | | | | | | |
| --- | --- | --- | --- | --- | --- | --- | --- | --- | --- | --- | --- | --- |
| Conventional job attributes | | | | | | | | | | | | |
| Working hours | | | 1  2 | | A position for a qualified nurse at a home care service is advertised.  full-time (40 hours per week)  part-time (20 hours per week). | | | | | | | |
| Wage | | | 1  2  3  4  5  6 | | The gross hourly wage without bonuses is  18 euros. The monthly salary is approximately 3,100 euros (full-time) / 1,550 euros (part-time) gross.  20 euros. The monthly salary is approximately 3,500 euros (full-time) / 1,750 euros (part-time) gross.  22 euros. The monthly salary is approximately 3,800 euros (full-time) / 1,900 euros (part-time) gross.  24 euros. The monthly salary is approximately 4,200 euros (full-time) / 2,100 euros (part-time) gross.  26 euros. The monthly salary is approximately 4,500 euros (full-time) / 2,250 euros (part-time) gross.  28 euros. The monthly salary is approximately 4,900 euros (full-time) / 2,450 euros (part-time) gross. | | | | | | | |
| Number of patients per nurse | | | 1  2  3 | | At this employer, the qualified nurses  are responsible for fewer patients compared to your current workplace. On a full workday, there are 2 fewer patients.  are responsible for the same number of patients as at your current workplace.  Are responsible for more patients compared to your current workplace. On a full workday, there are 2 more patients.  The effort per patient is expected to be similar to your current workplace. | | | | | | | |
| Family-friendly shift schedule | | | 1  2 | | Special consideration is given to family obligations (childcare, caregiving for relatives).  The wishes of all the nurses are equally considered. | | | | | | | |
| Childcare | | | 1  2 | | The employer provides childcare during working hours.  Employees with children at this employer must arrange for childcare themselves. | | | | | | | |
| Attributes of self-organizing nursing teams | | | | | | | | | | | | |
| Nursing autonomy | | | 1  2 | | Unlike usual practices, the nurses can decide for themselves which nursing services to provide. They are not bound by a fixed service catalog.  Nursing services are usually provided based on a fixed catalog. | | | | | | | |
| Participatory shift scheduling | | | 1  2 | | The schedule is set by the employer and is generally reliable.  The schedule is created with the involvement of all the nurses and is generally reliable. | | | | | | | |
| Hierarchy | | | 1  2 | | In the team  there is a clear hierarchy between management and nurses.  there is no noticeable hierarchy between management and nurses. | | | | | | | |
| Type of care documentation | | | 1  2 | | Documentation is done via entries on a smartphone.  Documentation is done using a voice app that automatically records spoken entries. | | | | | | | |
| **How attractive is this job for you?** | | | | | | | | | | | | |
| Very  unattractive | |  | |  | |  |  |  |  |  | Very  attractive | |
| 1 | 2 | 3 | | 4 | | 5 | 6 | 7 | 8 | 9 | 10 | 11 |
| ❑ | ❑ | ❑ | | ❑ | | ❑ | ❑ | ❑ | ❑ | ❑ | ❑ | ❑ |
| **How physically demanding is this job for you?** | | | | | | | | | | | | |
| Not very demanding | |  | |  | |  |  |  |  |  | Very demanding | |
| 1 | 2 | 3 | | 4 | | 5 | 6 | 7 | 8 | 9 | 10 | 11 |
| ❑ | ❑ | ❑ | | ❑ | | ❑ | ❑ | ❑ | ❑ | ❑ | ❑ | ❑ |
| **How mentally demanding is this job for you?** | | | | | | | | | | | | |
| Not very demanding | |  | |  | |  |  |  |  |  | Very demanding | |
| 1 | 2 | 3 | | 4 | | 5 | 6 | 7 | 8 | 9 | 10 | 11 |
| ❑ | ❑ | ❑ | | ❑ | | ❑ | ❑ | ❑ | ❑ | ❑ | ❑ | ❑ |

Note: The table provides an overview of the vignettes’ dimensions and levels. The right column lists all possible vignette configurations form top to bottom.
Source: Factorial survey of home care staff.

Figure A1: Graphical representation of main effects of job characteristics on job attractiveness, physical strain, and mental strain

**
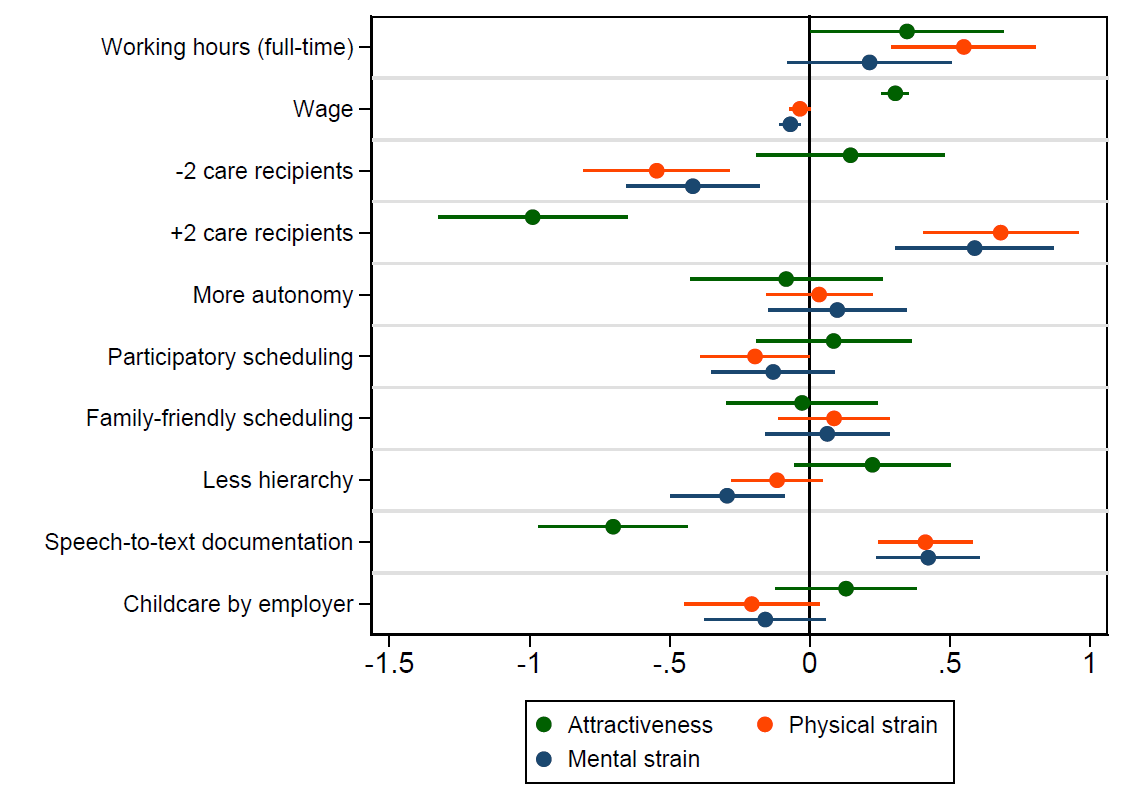
**

Note: Dots represent point estimates; horizontal lines indicate 90% confidence intervals. The x-axis shows effect sizes. Marker colors correspond to the three outcomes: attractiveness (green), physical strain (red), and mental strain (blue). Estimates are obtained from separate regression models for each outcome.
Source: Factorial survey of (former) home care staff, own calculations.

Figure A2: Graphical representation of average and subgroup treatment effects

|  |  | **Attractiveness** | | | | | | | | **Physical strain** | | | | | | | | **Mental strain** | | | | | | | | |
| --- | --- | --- | --- | --- | --- | --- | --- | --- | --- | --- | --- | --- | --- | --- | --- | --- | --- | --- | --- | --- | --- | --- | --- | --- | --- | --- |
|  |  | Whole Sample | Age | | Children <6 years | | | Gender | | Whole Sample | Age | | Children <6 years | | | Gender | | Whole Sample | Age | | | Children<6 | | | Gender | |
|  |  |  | young | old | no | yes | m | | f |  | young | old | no | yes | m | | f |  | young | old | no | | yes | m | | f |
| Demands | Alternative documentation (speech-to-text) # |  |  |  |  |  |  | |  |  |  |  |  |  |  | |  |  |  |  |  | |  |  | |  |
|  | Working hours (full-time) |  |  |  |  |  |  | |  |  |  |  |  |  |  | |  |  |  |  |  | |  |  | |  |
|  | Less patients |  |  |  |  |  |  | |  |  |  |  |  |  |  | |  |  |  |  |  | |  |  | |  |
|  | More patients |  |  |  |  |  |  | |  |  |  |  |  |  |  | |  |  |  |  |  | |  |  | |  |
| Resources | Autonomy # |  |  |  |  |  |  | |  |  |  |  |  |  |  | |  |  |  |  |  | |  |  | |  |
|  | (Clear) hierarchy # |  |  |  |  |  |  | |  |  |  |  |  |  |  | |  |  |  |  |  | |  |  | |  |
|  | Participatory shift scheduling # |  |  |  |  |  |  | |  |  |  |  |  |  |  | |  |  |  |  |  | |  |  | |  |
|  | Family-friendly shift scheduling |  |  |  |  |  |  | |  |  |  |  |  |  |  | |  |  |  |  |  | |  |  | |  |
|  | Childcare support by employer |  |  |  |  |  |  | |  |  |  |  |  |  |  | |  |  |  |  |  | |  |  | |  |
|  | wage |  |  |  |  |  |  | |  |  |  |  |  |  |  | |  |  |  |  |  | |  |  | |  |

Note: Green, beneficial effect; red, critical/potentially harmful effect; lighter colors, subgroup effects; white, no significant effect.
Source: Factorial survey of (former) home care staff, own calculations.
